# Screening with the Bilateral Corneal Symmetry 3-D Analyzer

**DOI:** 10.3390/ijerph22050747

**Published:** 2025-05-09

**Authors:** Shiva Mehravaran, Allen Eghrari, Siamak Yousefi, Fahmi Khalifa, Guita Ghiasi, Azadeh Farahi

**Affiliations:** 1 Department of Biology, School of Computer, Mathematical, and Natural Sciences, Morgan State University, Baltimore, MD 21251, USA; guita.ghiasi@morgan.edu; 2 Department of Ophthalmology, Johns Hopkins University School of Medicine, Baltimore, MD 21287, USA; allen@jhmi.edu; 3 Department of Ophthalmology and Department of Genetics, Genomics, and Informatics, University of Tennessee Health Science Center, Memphis, TN 38136, USA; siamak.yousefi@uthsc.edu; 4 Department of Electrical and Computer Engineering, School of Engineering, Morgan State University, Baltimore, MD 21251, USA; fahmi.khalifa@morgan.edu; 5 Noor Ophthalmology Research Center, Tehran P.O. Box 3475-19395, Iran; az_farahi@yahoo.com

**Keywords:** corneal topography, interocular symmetry, keratoconus, corneal elevation, anterior cornea, posterior cornea

## Abstract

This study aimed to evaluate the effectiveness of an innovative platform (the Bilateral Corneal Symmetry 3-D Analyzer—BiCSA) and a novel corneal symmetry index (the Volume Between Spheres—VBS) in differentiating normal corneas from those with keratoconus. Pentacam imaging data from 30 healthy corneas and 30 keratoconus cases were analyzed. BiCSA was utilized to determine the VBS for each case. Statistical analyses included comparing mean VBS values between groups and assessing sensitivity, specificity, and positive predictive values (PPVs). Keratoconus patients exhibited significantly higher VBS scores compared to healthy controls, particularly within the central 4.0 mm zone (11.4 versus 6.3). Using a VBS threshold of 11.3 in the central zone identified 40% of keratoconus cases (40% sensitivity), but 100% of cases surpassing the threshold were keratoconus (100% PPV). Lowering the threshold to 10.4 increased case detection to 90% while maintaining a high PPV (84.2%). These findings suggest that VBS, particularly when focused on the central 4.0 mm zone, can be a valuable tool for early keratoconus screening and identifying potential corneal abnormalities requiring further clinical evaluation. No healthy control corneas in this study exceeded a VBS threshold of 11.4 at 4 mm, indicating that values above this warrant further investigation.

## 1. Introduction

Corneal topography and elevation maps are fundamental tools in ophthalmology, providing crucial insights into corneal shape and curvature. While unilateral corneal assessments offer valuable information, recognizing and characterizing asymmetry between fellow eyes is gaining increasing clinical significance [1,2,3,4,5,6]. Current symmetry analyses often rely on a limited set of parameters, such as local curvature or single-point thickness measurements, which may fail to capture subtle but clinically relevant deviations.

To address this limitation, there is a critical need for advanced methodologies that comprehensively assess corneal symmetry across the entire surface. This necessitates the development of novel automated algorithms for generating symmetry colormaps, systematically classifying their patterns, and establishing robust metrics and reference ranges for detecting abnormal corneas.

**Figure 1:**
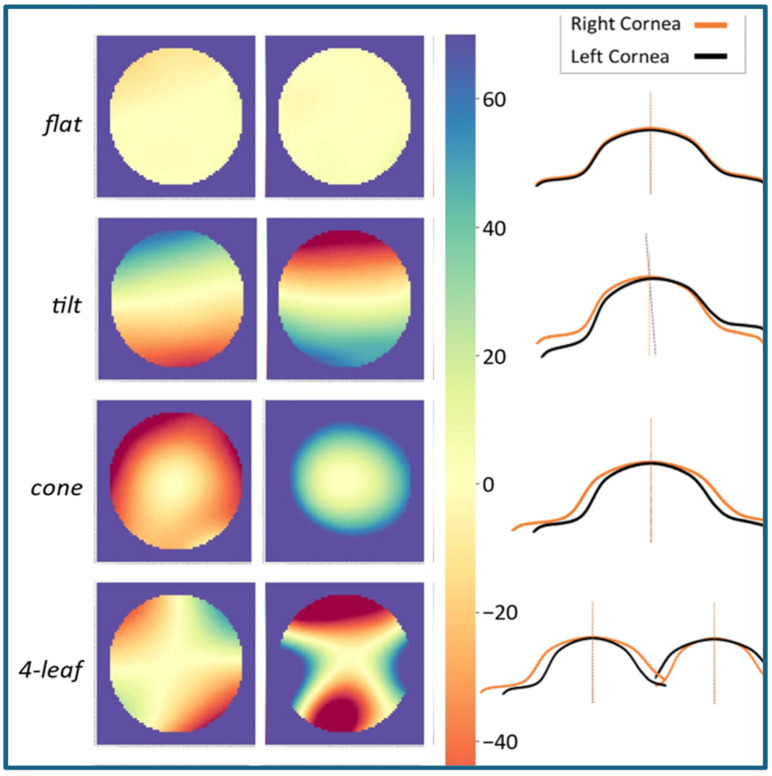
Representative examples of corneal symmetry maps. The “flat” pattern is characteristic of high symmetry observed in healthy corneas, “tilt” is potentially indicative of imaging or visual axis discrepancies, the “cone” pattern is consistent with the presence of keratoconus, and the “4-leaf” pattern is potentially associated with aniso-astigmatism or direct symmetry in the presence of corneal astigmatism.

In our previous work [7], we explored the feasibility of utilizing the fellow eye as a reference surface to analyze elevation symmetry across the entire cornea. Using a dataset of 4613 participants with Pentacam images, we developed a method for subtracting elevation matrices, generating color-coded symmetry maps, and extracting key features for machine learning-based analysis. This analysis revealed distinct anterior elevation symmetry patterns, including the “flat” pattern indicative of high symmetry in normal corneas, the “tilt” pattern potentially associated with imaging or visual axis discrepancies, the “cone” pattern consistent with keratoconus, and the “4-leaf” pattern potentially related to aniso-astigmatism or direct symmetry in the presence of astigmatism. These patterns are further illustrated in Figure 1.

To translate these findings into clinical practice, we have developed a dedicated software tool (the Bilateral Corneal Symmetry 3-D Analyzer—BiCSA version 9.1.0.0) that automates the analysis of corneal symmetry. BiCSA incorporates advanced image registration techniques, developed using machine learning, to correct for potential head tilts or rotations during image acquisition. This software compares thousands of anterior and posterior corneal elevation measurement points between fellow eyes, providing quantitative metrics of symmetry and identifying unique patterns of asymmetry.

By leveraging this innovative tool and its integrated image registration capabilities, we aim to enhance the detection of subtle corneal abnormalities, particularly those that are undetectable by conventional unilateral assessments. In our current studies, the aim is to assess the performance of BiCSA alongside a novel corneal symmetry index, the Volume Between Spheres (VBS). VBS quantifies the space enclosed between two spheres, where one cornea is centered on its fellow cornea, offering a unique measure of inter-eye symmetry and asymmetry. The primary aim of this preliminary investigation was to assess the feasibility and performance of the VBS metric in differentiating keratoconus from normal corneas for screening purposes.

## 2. Materials and Methods

In this retrospective study, we analyzed a subset of Pentacam imaging data from 60 eyes from 30 patients with healthy corneas and 30 patients diagnosed with keratoconus. Inclusion criteria for the healthy group included normal visual acuity, no history of ocular surgery, and no evidence of corneal disease based on clinical examination and Pentacam topography maps. Inclusion criteria for the corneal disease group included a clinical diagnosis of corneal disease by a corneal specialist and no history of ocular surgery.

Imaging had been performed on all participants using the Pentacam HR (Oculus Optikgeräte GmbH, Wetzlar, Germany). Each Pentacam scan generates a 140 × 140 matrix of elevation data points, representing approximately 20,000 elevation measurements across each of the anterior and posterior corneal surfaces. The BiCSA was utilized to analyze the Pentacam data and derive the VBS index for each case.

The BiCSA software input is the 140 × 140 raw data matrices of corneal surface elevation measurements for the left and right fellow eyes, as measured by and exported from the Pentacam. The data matrices from the two eyes are initially aligned by matching their centers and corresponding points, establishing a consistent reference for comparison. The software then applies iterative image registration techniques to refine the alignment between the two matrices. The goal at this point is to account for the type of symmetry (i.e., mirror versus direct symmetry) and head tilt or rotation in different planes (differences in head positioning during the imaging of each eye).

At each iteration, a point-by-point subtraction is performed, subtracting each data point in the right-eye matrix from the corresponding point in the registered left-eye matrix. The resulting difference matrix represents elevation differences between the two corneas. To ensure that positive and negative differences did not cancel each other out, the absolute values of the elevation differences are calculated within the difference matrix during each iteration. The process iterates until the optimal adjustment (minimum VBS) is achieved.

The key adjustments are as follows:

Flip: For mirror symmetry, the matrix for the left eye is flipped along the y-axis. This adjustment accounts for the natural mirror image relationship between fellow eyes versus direct symmetry.

Shift: Positional adjustments can be applied along the x, y, and z axes. These shifts correct for slight discrepancies in central points of the matrices in reference to the corneal center.

Rotate: A rotational adjustment can be made to align the orientation of the two images. This correction addresses different head positions along the frontal plane during the imaging of each eye.

Tilt: A tilt correction addresses angular misalignment caused by differences in head positioning during imaging along the sagittal or transverse planes.

These adjustments can be made either manually or automatically. In the “manual” mode, the user can change the values for each of these parameters and see how these adjustments change the difference map and VBS value. In the “auto” mode, these adjustments are performed iteratively, with the software refining the alignment at each step to minimize the VBS value.

While Figure 1 displays profile lines that offer a 2D representation of these inter-eye elevation differences, readers should note that VBS calculations occur in full 3D space, integrating elevation disparities across the entire corneal dome rather than just along a single 2D cross-section.

Finally, the average of these absolute elevation differences is calculated within the defined zone (as selected by the user). The VBS index represents the average of all absolute elevation differences within a given zone. For this study, the 6.0 mm and 4.0 mm zones were used.

Statistical analysis was performed using Microsoft Excel. Mean VBS values were calculated for each group, and an independent samples *t*-test was used to compare mean VBS values between groups. We also determined the sensitivity, specificity, and positive predictive value (PPV) for different VBS thresholds.

The study was conducted according to the guidelines of the Declaration of Helsinki and approved by the Institutional Review Board of Morgan State University (IRB #20/10-0119, approved on 30 September 2020).

## 3. Results

### 3.1. Examples of BiCSA Output

#### 3.1.1. Normal Cornea

**Figure 2:**
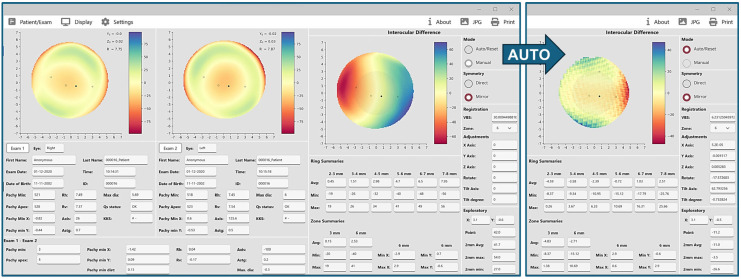
Anterior elevation symmetry maps before and after auto-registration in a normal case. In this example, the right-hand panel in the screenshot on the left indicates that alignment is on the manual mode, mirror symmetry, and no adjustments to registration parameters. According to the ring and zone summary data, the colors of the difference map, and the Volume Between Spheres (VBS), interocular difference is relatively high, and corneal pathology or abnormality can be suspected. However, the VBS value is significantly reduced from 30 to about 6 after applying auto-alignment and registration (far right), and the map pattern becomes “flat” pattern, which is indicative of normal corneas bilaterally.

Figure 2 illustrates the impact of image registration in a case with normal corneas. Initially, alignment was performed with no image adjustments, resulting in a “tilt” symmetry pattern and a high VBS value (30 units) suggesting potential pathology. However, after applying automated image registration, the VBS value was significantly reduced to approximately 6 units, and the symmetry map transitioned to a “flat” pattern, characteristic of highly symmetric bilateral normal corneas (Figure 1).

#### 3.1.2. Aniso-Astigmatism

**Figure 3:**
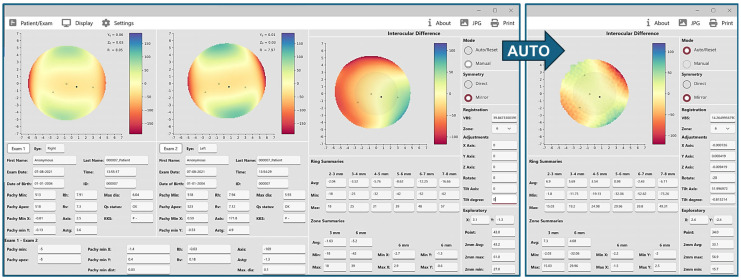
Anterior elevation symmetry maps before and after auto-registration in a case of aniso-astigmatism. In this example, alignment is first on the manual mode with mirror symmetry and no adjustments to registration parameters. According to summary data, the colors and map, and the Volume Between Spheres (VBS), the interocular difference is relatively high, and the presence of a corneal pathology or abnormality can be suspected. However, after applying auto-alignment and registration (far right), interocular differences are significantly smaller, VBS is reduced by 26 units (from 40 to 14), and the map pattern becomes “four-leaf”, which is consistent with aniso-astigmatism. The bottom left panel of the left screenshot (Exam 1–Exam 2) also shows an interocular difference of 1.3 diopters of astigmatism.

Figure 3 illustrates anterior elevation asymmetry maps in a case of aniso-astigmatism. Initial manual alignment with no image registration revealed an irregular symmetry pattern with a high VBS value. After applying automated image registration, the interocular differences were significantly reduced, the VBS decreased from about 40 to approximately 14, and the symmetry map transitioned to a “4-leaf” pattern, consistent with aniso-astigmatism.

### 3.2. Keratoconus Detection

#### 3.2.1. Group Comparisons

Analysis of anterior corneal elevation asymmetry within the central 4.0 mm zone revealed a significant difference between patients with keratoconus and healthy controls (independent *t*-test *p* < 0.0001). The mean VBS score was 6.3 in the healthy control group, and patients with keratoconus demonstrated a significantly higher mean VBS score of 11.4 and a mean inter-group difference of 5.1 units (95% confidence interval of mean difference: 2.2 to 8.1). This represents a strong association between increased corneal asymmetry and the presence of keratoconus. Comparison VBS scores for the posterior 4.0 mm of the control and keratoconus cohorts revealed a mean difference of 5.7 units in the setting of keratoconus (14.5 vs. 20.2, *p* = 0.02, 95% CI: 0.85 to 10.6).

When assessing asymmetry within a larger 6.0 mm zone, the difference between groups was less pronounced (mean difference of 3.4 units, *p* = 0.11), suggesting that the most significant asymmetry in keratoconus manifests in the central corneal region.

#### 3.2.2. Sensitivity and Specificity Analysis

For keratoconus screening, a VBS threshold of 11.3 in the central 4.0 mm zone yielded 100% PPV and identified 40% of cases. Lowering the threshold to 10.4 increased case detection to 90% while maintaining a high PPV (84.2%).

**Figure 4:**
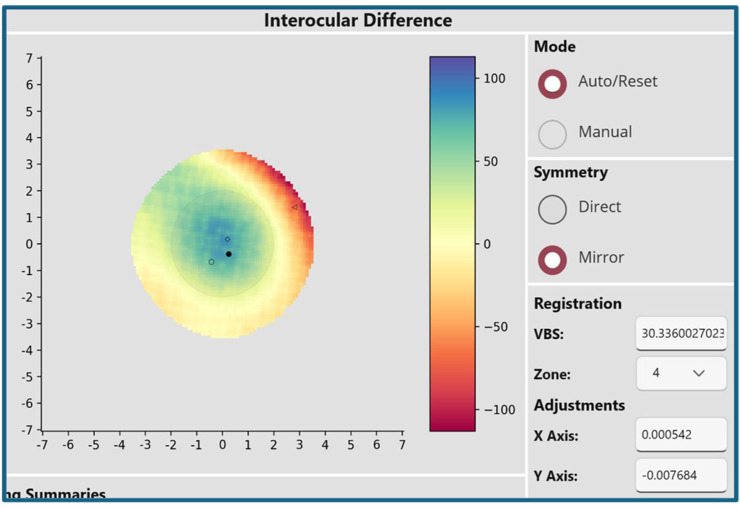
Anterior elevation symmetry map in a case of keratoconus. In this example, the Volume Between Spheres score of 30.3 significantly exceeds the established cutoffs of 10.4 and 11.3. The central blue area in this map represents the region of greatest asymmetry between fellow eyes, consistent with the characteristic bulging observed in keratoconus.

Figure 4 illustrates a case of keratoconus where the anterior elevation VBS score is 30.3, far exceeding the cutoffs of 10.4 or 11.3. The blue area appreciated in the center of the image reflects the asymmetry in how the central cornea of the left eye is bulging forward.

## 4. Discussion

Numerous studies have demonstrated the effectiveness of machine learning and deep learning models in detecting keratoconus using various corneal parameters [8,9,10,11,12,13]. More recent research has utilized a new corneal biomechanical parameter (the Corvis Biomechanical Index—CBI), which combines corneal thickness profiles with deformation parameters for improved keratoconus detection [14]. Additionally, some studies have employed deep learning to detect keratoconus using corneal dynamic videos [15]. While these approaches focus on specific corneal parameters or utilize advanced machine learning techniques, this study explores a novel approach: machine-learning-based analysis of interocular corneal symmetry using data from the entire corneal surface. This approach offers a more comprehensive assessment by considering the subtle differences between fellow eyes, enabling the detection of various corneal abnormalities beyond keratoconus through a single corneal imaging evaluation.

The BiCSA demonstrates significant potential for enhancing the clinical evaluation of corneal health. By quantifying interocular corneal symmetry into various metrics and generating comprehensive symmetry maps, BiCSA provides valuable insights beyond traditional unilateral assessments.

The ability to identify distinct symmetry patterns—such as the “flat” pattern in healthy corneas and the “cone” pattern associated with keratoconus—highlights BiSCA’s capability to characterize corneal morphology with greater precision. Furthermore, integrating advanced image registration techniques significantly enhances the accuracy and reliability by correcting for potential head tilts and/or rotations during image acquisition.

The successful identification of keratoconus cases, with high sensitivity and specificity at optimized VBS thresholds, underscores the clinical utility of BiCSA for early disease detection. Importantly, BiCSA may also prove valuable in identifying subtle corneal abnormalities that might be missed in routine clinical examinations.

### 4.1. Clinical Applications

#### 4.1.1. Anterior Corneal Conditions

In clinical practical use, this technique can flag abnormalities beyond keratoconus for eye care professionals to examine, as various conditions may affect the anterior cornea. Given that no control corneas in this sample exceeded the VBS threshold of 11.4 at 4 mm, values higher than this can be marked for further review.

Traditional corneal evaluations often fail to detect subtle abnormalities, particularly those located in the corneal periphery, as they primarily focus on the central region. For example, conditions like anterior basement membrane dystrophy with Salzmann Nodular Degeneration (SND) can develop subtle peripheral nodules that remain undetected during routine exams. SND is characterized by the formation of gray-white to bluish nodules, which are more commonly located in the peripheral cornea [16]. While these nodules may not initially impair central vision, they can progressively extend toward the central cornea, causing irregular astigmatism and visual distortion. Symmetry analysis between fellow eyes, as performed by BiCSA, can be a valuable tool for identifying SND earlier and more easily. By comparing the topographical maps of both eyes, clinicians can detect asymmetries or abnormalities that might not be apparent when examining each eye individually. This enhanced detection capability allows for earlier diagnoses and interventions, potentially improving patient outcomes.

#### 4.1.2. Posterior Corneal Conditions

Beyond anterior corneal conditions, BiCSA’s diagnostic capabilities may have significant implications for posterior corneal disease management. Fuchs dystrophy, a progressive condition characterized by corneal endothelial cell dysfunction, is a leading cause of corneal transplantation. While current clinical assessments can identify Fuchs dystrophy, predicting disease progression and the need for future intervention remains challenging [17]. BiCSA’s analysis of posterior corneal elevation may provide valuable prognostic information. For example, in our preliminary analysis, patients with Fuchs dystrophy who exhibited higher VBS values for posterior corneal elevation were more likely to require subsequent corneal transplantation.

## 5. Conclusions

To our knowledge, BiCSA represents a unique and novel approach to corneal symmetry analysis, offering a comprehensive and automated platform for assessing corneal health, identifying subtle abnormalities that may go undetected in routine examinations, and decision making for timely ophthalmic interventions. To address the limited sample size in this preliminary analysis, currently, we are conducting a larger study with 300 keratoconus cases and 300 normal cases. This expanded study stratifies keratoconus cases by disease severity in pairs of eyes (e.g., normal–mild KCN, subclinical–mild, and normal–subclinical, etc.), allowing for a more nuanced evaluation of the VBS metric across varying stages of keratoconus. Further research and clinical validation are crucial to fully explore the clinical potential of BiCSA in various corneal conditions. Suggestions include the following areas:

1. Feature Integration: By integrating the VBS index with additional corneal parameters (e.g., curvature metrics, pachymetry indices, and posterior elevation data), machine learning models could uncover nonlinear relationships and subtle patterns that might enhance sensitivity.
2. Training on Diverse Data: Training AI models on larger and more diverse datasets, such as the ongoing study involving 300 keratoconus and 300 normal cases, and hopefully a clinical dataset of 1000 s of patients (currently discussing collaborations) would allow for a more accurate identification of keratoconus across its full spectrum.
3. Threshold Optimization: Machine learning techniques could optimize classification thresholds dynamically, striking a balance between sensitivity and specificity based on clinical priorities.

## 6. Patents

The methods and systems described herein are the subject of a published patent application (US20240074654A1) [18].

## Data Availability

The data that support the findings of this study are available from the corresponding author upon reasonable request. Researchers interested in utilizing the BiCSA software for their own research are encouraged to contact the corresponding author to discuss potential collaborations.

## Abbreviations

The following abbreviations are used in this manuscript:

BiSCA	The Bilateral Corneal Symmetry 3-D Analyzer
VBS	Volume Between Spheres
PPV	Positive Predictive Value
